# Comments on ‘Hydrogen bonds in crystalline d-alanine: diffraction and spectroscopic evidence for differences between enantiomers’

**DOI:** 10.1107/S2052252518007406

**Published:** 2018-07-27

**Authors:** Hans-Beat Bürgi, Piero Macchi

**Affiliations:** a Department of Chemistry and Biochemistry, University of Bern, Freiestrasse 3, Bern, 3012, Switzerland; b Department of Chemistry, University of Zurich, Winterthurestrasse 190, Zurich, 8057, Switzerland

**Keywords:** parity-violation energy, enantiomers, phase transitions, amino acids

## Abstract

The recent paper by Belo, Pereira, Freire, Argyriou, Eckert & Bordallo [(2018 ▸), *IUCrJ*, **5**, 6–12] reports observations that may lead one to think of very strong and visible consequences of the parity-violation energy difference between enantiomers of a molecule, namely alanine. If proved, this claim would have an enormous impact for research in structural chemistry. However, alternative, more realistic, explanations of their experiments have not been ruled out by the authors. Moreover, the theoretical calculations carried out to support the hypothesis are unable to differentiate between enantiomers (molecules or crystals). Therefore, the conclusions drawn by Belo *et al.* (2018 ▸) are deemed inappropriate as the data presented do not contain sufficient information to reach such a conclusion.

## Abstract 2

The recent claim of structural differences between alanine enantiomers in the solid state is critically discussed.

## 1. Introduction   

In a recent paper, entitled ‘Hydrogen bonds in crystalline d-alanine: diffraction and spectroscopic evidence for *differences between enantiomers*’ (our emphasis), Belo *et al.* (2018 ▸) report polarized Raman spectra collected on hydrogenated d-alanine single crystals (C_3_H_7_NO_2_, d-ala-h_7_), neutron powder diffraction (NPD) measurements on fully deuterated d-ala-d_7_ and *ab initio* calculations of the harmonic vibrational frequencies of an isolated d-ala molecule and of d- and l-ala crystals. In the abstract of the paper, the authors conclude that their ‘results reveal *dissimilarities in the structural properties of d-alanine compared with l-alanine*’ (our emphasis). These are very strong statements. In their generality, they are no less than a refutation of the empirically and theoretically founded principle that enantiomeric molecules have the same energies and the same chemical properties; optical activity and circular dichroism are the same in absolute value but have opposite sign.

The present doctrine derives from the Schrödinger equation with a Hamilton operator accounting for electromagnetic forces. This theory has not only been shown to explain successfully and quantitatively all kinds of experimental chemical and physical results, it is also parity invariant, *i.e.* its mathematical structure requires that the energies of enantiomers be identical; their equilibrium structures and their potential energy surfaces must be exact mirror images of each other and their vibrational spectra identical (Quack, 2014 ▸). Differences between enantiomeric molecules are only possible in a theory that violates parity, *i.e.* a theory that accounts for the weak nuclear force, the only kind of force that breaks parity (see, for example, Quack *et al.*, 2008 ▸). Belo *et al.* (2018 ▸) allude to this possibility in the introduction to their report without mentioning, however, that energy differences between enantiomers due to parity violation (PV) are extremely small. For d- and l-ala, differences of the order of 10^−14^ kJ mol^−1^ have been calculated in both the gaseous and aqueous phases and have been found to depend on conformation, *i.e.* for some conformations d-ala is more stable, for others l-ala is more stable (Laerdahl *et al.*, 2000 ▸; Berger & Quack, 2000 ▸; Quack, 2014 ▸). On the basis of such calculations, Quack and collaborators estimated that an experimental verification of these differences by vibrational spectroscopy of suitable molecules would require a spectral resolution Δν_PV_/ν of the order of 10^−16^ to 10^−19^ (Quack *et al.*, 2008 ▸), a value which has not yet been reached with present day technology (Albert *et al.*, 2017 ▸). It is therefore highly unlikely that PV affects molecular and crystal structures as well as their energies and vibrational spectra in a way that is observable from present day diffraction and spectroscopic experiments.^1^

If correct, the far-reaching interpretation of their experimental data by Belo *et al.* (2018 ▸) represents a refutation of the basic tenet implicit in the usual quantum chemical Schrödinger equation that currently represents so-called ‘normal science’ according to Kuhn (1962 ▸). It could thus represent the beginning of a scientific revolution that might lead to the formulation of a new paradigm (and perhaps a revision of the currently accepted values of parity-violation energy) and, by accumulation of additional evidence, to a new ‘normal science’. Given the potential consequences of such events, it is mandatory to confirm the new evidence with every imaginable and feasible control experiment and to eliminate conventional explanations of the new evidence as far as possible. We comment on the paper by Belo *et al.* (2018 ▸) with these thoughts in mind.

## 2. Some general comments on comparing and interpreting data of enantiomers   

Our comments are guided by five main questions:

### 2.1. Are all data available for both enantiomers?   

If not, any observation judged unusual cannot necessarily be attributed to a difference between enantiomers, as the same or a similar observation might be made for the opposite enantiomer as well. We note that the Raman scattering data for d-ala-h_7_ reported by Belo *et al.* (2018 ▸) are not matched with correspondingly detailed data for l-ala-h_7_. Thus, it cannot be excluded that any ‘unusual’ observation in one enantiomer might also be found in the other one, potentially making the two enantiomers the same.

### 2.2. Have alternative explanations, not related to the putative phase transition associated with the Salam hypothesis on interconversion between enantiomers, been considered for ‘unusual’ observations in only one enantiomer?   

In the early 1990s, Salam (1991 ▸, 1992 ▸) suggested that parity violation may imply a second-order phase transition below a critical temperature involving tunnelling of the less stable into the more stable enantiomer. Subsequently, several authors reported observations that were interpreted as evidence supporting Salam’s hypothesis (*e.g.* Wang *et al.*, 2002 ▸; Belo *et al.*, 2018 ▸) without excluding alternative explanations for their observations. Some conventional explanations of the putative unusual phenomena observed in the Raman data for d-ala-h_7_ are suggested below in Section 3.2.

### 2.3. If data for both enantiomers are compared, are the histories of the respective samples and their chemical analysis the same?   

The powder diffraction data on d-ala-d_7_ (Belo *et al.*, 2018 ▸) and l-ala-d_7_ (De Souza *et al.*, 2009 ▸) come from two experiments published ∼10 years apart. There is no comparison, neither of the histories of the two samples nor of their analytical data, *e.g.* the H/D ratios in the recrystallized samples. Even though deuterated water was used for the recrystallization of deuterated samples, one cannot exclude exchange of D for H, especially at the ND_3_ group, unless the recrystallizations were carried out in a dry atmosphere. Sullivan *et al.* (2003 ▸) noticed that the heat of transition associated with a signal in the *C*_p_ *versus T* specific heat curve of l-ala-h_7_ around 270 K decreased as the number of crystallization cycles increased. This is clear evidence for a history dependence of some sample properties. By ‘history of the sample’, we mean a number of features that depend on the treatment of the species before and after the preparation of crystals used for data collection (*e.g.* purity, degree of crystallinity, grain size, type and number of defects). The effects of such a dependence on the properties reported by Belo *et al.* (2018 ▸) have to be excluded before the data from two different samples can be compared conclusively.

### 2.4. Have alternative explanations been considered for differences between enantiomers, *i.e.* explanations not related to the putative phase transition associated with the Salam hypothesis?   

Belo *et al.* (2018 ▸) reported significant differences between l-ala and d-ala in the positions of the D atoms of the ammonium groups refined from the NPD data and, consequently, different geometries of the D⋯O hydrogen bonds. We note that they do not report refinement of the powder data of d-ala-d_7_ starting from the structure model obtained from the l-ala-d_7_ powder data and *vice versa*; multiple minima in the crystallographic least-squares surface have thus not been excluded. Such an experiment would be particularly important with powder data, given their restricted information content compared with single-crystal data.

### 2.5. How do the postulated differences between enantiomers compare with the present state of quantum theory?   

The periodic density functional theory (DFT) calculations of inelastic neutron scattering (INS) spectra for d- and l-ala-h_7_ discussed at the end of Section 3.1 of Belo *et al.* (2018 ▸) are said to show differences between l-ala and d-ala in the optimized geometries, in particular the N—H optimized distances. In keeping with this, the calculated inelastic neutron scattering of the two enantiomeric crystals differ as well. However, Belo *et al.* (2018 ▸) used model Hamiltonians containing only the potentials of electromagnetic forces (GGA DFT + Vanderbilt ultra-soft pseudopotential). The weak forces that violate the parity are not included, therefore effects of PV cannot emerge from these calculations. A possible explanation for the findings of Belo *et al.* (2018 ▸) is suggested in Section 3.3.

## 3. Some specific comments on comparing and interpreting data from d- and l-ala   

### 3.1. Data from the literature   

It is certainly true, as is also mentioned by Belo *et al.* (2018 ▸), that l-ala has been studied intensively as a function of temperature or pressure by both Raman spectroscopy and X-ray and neutron diffraction. Some diffraction data sets show very high resolution and have been collected at very low temperature, as required for accurate charge-density determinations. By comparison, d-ala has been investigated much less (it is more expensive!), mainly with the intention of finding experimental confirmation of the effects of PV. While Wang *et al.* (2002 ▸) claimed that differences exist between the enantiomers, Sullivan *et al.* (2003 ▸) found no unusual behaviour in their X-ray diffraction and NMR experiments in the temperature range expected for the putative phase transition (∼270 K). They also presented arguments against the Salam hypothesis for the molecules under study. Wilson *et al.* (2005 ▸) could offer no structural support of the Salam hypothesis based on single-crystal neutron diffraction studies of d-ala-h_7_ and l-ala-h_7_ at 60 K and room temperature.

Note that all single-crystal neutron diffraction experiments on both d-ala and l-ala have been performed with hydrogenated species (Wilson *et al.*, 2005 ▸; Lehmann *et al.*, 1972 ▸), whereas the powder diffraction data used by Belo *et al.* (2018 ▸) come from deuterated species, for both d- and l-ala. One might therefore be tempted to conclude that the putative PV effects occur for ala-d_7_ only and not for ala-h_7_. However, differences between hydrogenated and deuterated d- or l-ala have not been investigated with experiments of comparable accuracy, neither neutron single-crystal diffraction nor NPD for both isotopomers. This prevents a conclusive comparison between the isotopomers.

### 3.2. Unusual Raman spectroscopic behaviour of one enantio­mer   

The Raman studies concentrate on ‘anomalies in the lattice modes of hydrogenated d-ala’ (Section 3.1 of Belo *et al.*, 2018 ▸). Two of the anomalies mentioned are the appearance of ‘new bands’ and ‘bands that split at lower temperatures’ (caption to Fig. 2 of Belo *et al.*, 2018 ▸). One of these new bands (at ∼100 cm^−1^ in Fig. 2*a* of Belo *et al.*, 2018 ▸) is indicated to appear at and below 208 K. Inspection of the figure suggests that the band is present all the way to 300 K as a shoulder of the very strong signal at ∼113 cm^−1^. Another such band is said to appear at ∼170 cm^−1^ below 175 K. Both of them are identified as *B* bands appearing in the *A*-band spectrum with small intensities. In Fig. 3(*a*), which shows the *B* bands, these signals are seen at all temperatures between 21 and 290 K. The one at 170 cm^−1^ shifts to lower frequency at the higher temperatures, reduces its maximal intensity and becomes broader. The behaviour of this band in the *A* spectrum is not incompatible with its behaviour in the *B* spectrum. Since the experimental part says nothing about the accuracy of the crystal orientation relative to the probing laser beam, it cannot be excluded that the *B* bands in the *A* spectrum are due to slight misorientation of the crystal. Such an explanation would make the postulated phase transition unnecessary, but is not considered.

Figs. 2(*b*) and 2(*c*) (Belo *et al.*, 2018 ▸) are said to indicate splitting of the bands at ∼140 cm^−1^ and ∼138 cm^−1^, respectively, observed at 283 K. The former slowly shifts position on cooling, until at 22 K it is found at ∼150 cm^−1^. From 160 K up it slowly merges with the band at 140 cm^−1^, which is still visible as a shoulder at 160 K and becomes accidentally degenerate with the shifting band at 283 K. The band at ∼138 cm^−1^ shows similar behaviour (Fig. 2*c* of Belo *et al.*, 2018 ▸), with the two bands visible to at least 208 K. These shifts indicate noticeable Grüneisen-type anharmonicity, *i.e.* a decrease in frequency with increasing crystal volume due to thermal expansion (Grüneisen, 1926 ▸; Kolesov, 2017 ▸). Such anharmonicity has also been deduced from the thermal evolution of atomic displace­ment parameters, which are mainly determined by the external lattice modes (Bürgi *et al.*, 2000 ▸; Aree *et al.*, 2014 ▸; the latter paper and its two predecessors discuss the closely related α-, β- and γ-glycine polymorphs). These observations suggest that the two bands seen at low temperatures persist all the way to room temperature, with the higher-energy band at ∼150 cm^−1^ shifting to smaller frequencies due to crystal expansion. Analogous arguments apply to the splittings discussed in Fig. 3 of Belo *et al.* (2018 ▸). Note that the alternative interpretation given here does not require a phase transition.

We postulate that the few examples of alternative explanations of the Raman scattering data by Belo *et al.* (2018 ▸) as given above – while not necessarily correct – would have had to be explicitly excluded before claiming – if only implicitly – a phase transition related to the Salam hypothesis and thus claiming ‘structural dissimilarities’ between enantiomers. Furthermore, a similarly detailed discussion of and comparison with corresponding data for l-ala-h_7_ is lacking.

### 3.3. Comments on theoretical calculations   

Belo *et al.* (2018 ▸, p. 10) state ‘A most noteworthy difference in the low-frequency dynamics of d- and l-ala is apparent when comparing the calculated 10 K INS spectra for l-ala *versus* d-ala, Fig. 5(*b*). It is quite obvious that there are significant differences in the vibrational amplitudes (*i.e.* peak intensities) of the low-frequency modes below 350 cm^−1^’. As mentioned in Sections 1 and 2.5, the DFT calculations by Belo *et al.* cannot account for PV since they do not contain the corresponding operator. In the context of differences between enantiomers this evidence is meaningless.

A possible explanation of their results – one that can be tested easily – might be as follows. Starting from their neutron powder structures for d- and l-ala, Belo *et al.* (2018 ▸) have optimized the respective atomic positions by DFT calculations and obtained different results for d- and l-ala. There is no mention of the energy difference between the two, nor of that between the structure optimized for d-ala and the inverted DFT-optimized structure of l-ala and *vice versa* (nor of the transition state energy between the two optimized structures, see Sullivan *et al.*, 2003 ▸). Could it be that the difference is a result of incomplete structure optimization of the different starting structures due to the convergence criteria incorporated in the DFT procedure used?

## 4. Conclusion   

Based on the comments above we conclude that Belo *et al.* (2018 ▸) have not presented coherent and conclusive ‘diffraction and spectroscopic evidence for differences between enantiomers’. Our conclusion concurs with those arrived at in earlier experimental and computational work (Berger & Quack, 2000 ▸; Laerdahl *et al.*, 2000 ▸; Sullivan *et al.*, 2003 ▸; Wilson *et al.*, 2005 ▸; Albert *et al.*, 2017 ▸) and with the current state of quantum chemical theory, including the effects of parity violation (Quack, 2014 ▸).

In the list below, we suggest some alternative explanations for the reported differences between d-ala and l-ala [(*a*)–(*c*)], and tests to confirm or exclude them [(*d*), (*e*)]:

(*a*) Incongruent crystallization processes for the enantiomeric substances, possibly leading to differences in sample characteristics, specifically the degree of deuteration or the density of crystal defects. Differences in sample treatment have been shown to explain differences observed in scanning temperature experiments (Sullivan *et al.*, 2003 ▸).

(*b*) Anharmonicity and isotope effects.

(*c*) Inconsistent structural optimization by the PV-free quantum chemical DFT method used.

(*d*) Comparison of Raman data for d- and l-ala.

(*e*) Tests for multiple structural minima during the refinement of NPD data.

We do not deny that the differences observed by Belo *et al.* (2018 ▸) are real. However, whatever they are, they have to be tested as suggested above before they can be attributed to ‘differences between enantiomers’.

*Note added in proof*: After submission of this work we became aware of similar work on l-nucleic acids [‘First look at RNA in l-configuration’ (Vallazza *et al.*, 2004 ▸) and ‘First experimental evidence for the preferential stabilization of the natural d- over the non-natural l-configuration in nucleic acids’ (Bolik *et al.*, 2007 ▸)]. The comments given above on the interpretation of differences between experimental data on enantiomers apply *a fortiori* to this work. Enantiomeric biomolecules such as duplex RNA octamers are even more difficult to characterize and compare than the relatively simple ala crystals.

## Footnotes

1 The articles by Quack reference much of the recent work by other researchers in this area.
